# Exposure Assessment of Nitrofuran Metabolites in Fish and Honey Produced in Armenia: A Pilot Investigation

**DOI:** 10.3390/foods12183459

**Published:** 2023-09-16

**Authors:** Davit Pipoyan, Meline Beglaryan, Victoria Chirkova, Alberto Mantovani

**Affiliations:** 1 Center for Ecological-Noosphere Studies, NAS RA, Abovyan Street 68, Yerevan 0025, Armenia; david.pipoyan@cens.am (D.P.); victoria.chirkova@cens.am (V.C.); 2 Italian National Food Safety Committee, Lungotevere Ripa 1, 00153 Rome, Italy; alberto.mantovani1956@gmail.com

**Keywords:** nitrofurans, honey, fish, risk, intake assessment, veterinary drugs

## Abstract

In Armenia, the presence of nitrofuran residues in food products is unacceptable for both domestic sales and export. However, food may contain nitrofuran metabolites (NMs) due to the illegal use of these drugs in the agrofarming practice. This study aimed to identify NMs as the marker residues for nitrofurans in fish and honey produced in Armenia and assess the potential health risks associated with consuming these foods. The commodities studied were natural honey and three species of farmed fish produced by various regions nationwide. Concentrations of the marker metabolites (3-amino-2-oxazolidinone (AOZ), 3-amino-5-methylmorpholino-2-oxazolidinone (AMOZ), 1-aminohydantoin (AHD), and semicarbazide (SEM)) were determined through an enzyme-linked immunosorbent assay (ELISA) and verified using liquid chromatography–mass spectrometry (LC-MS/MS). Consumer groups were identified based on their average daily intake of foods. Health risk was assessed by calculating the margin of exposure (MOE). Reference values for health risk assessment were obtained from the European Food Safety Authority (EFSA). Results showed that 33.3% of fish samples and 44.4% of honey samples contained NMs, the mean concentrations ranging from 0.05 μg/kg to 0.52 μg/kg. All MOE values obtained were over 10,000, indicating that the detected concentrations of NMs in fish and honey produced in Armenia pose no health risk to consumers. However, these results highlight the illicit use of highly toxic substances and the need for improved control of farming practices.

## 1. Introduction

The presence of residues of veterinary drugs (VD) in food products is a major concern in food safety, leading to strict requirements being set at both the national and international levels [1,2,3]. The prohibited agents may enter food products through various routes, such as illicit treatments or contaminated feed for food-producing animals [3,4]. 

In the 1980s, there was a growing global awareness of the threat posed by inadequately regulated veterinary drugs (VDs), such as antimicrobials used in animal husbandry. In response, many countries implemented measures to reduce the use of antimicrobials and prevent their potential adverse effects. Notable examples include Norway, which achieved a significant reduction of antibiotics in aquaculture by tenfold in 1987 through the application of vaccines. This reduction was accompanied by establishing maximum residue levels (MRLs) for substance residues in raw materials and food products and the requirement for veterinary drug sales by prescription from a veterinarian. Numerous countries across the globe supported the fight against antibiotics, with Japan reducing the use of antibiotics in livestock production by nearly 30% from 2000 to 2013 through the reduction of antibiotic tolerance and the prohibition of antimicrobial additives. South Korea also made significant progress, reducing antibiotic use by over a third between 2007 and 2016 by allowing the sale of antimicrobials only with veterinarian prescriptions [5,6,7].

The European Union has implemented comprehensive regulations that cover the entire food chain, from feed resources (Regulation (EC) No 1831/2003) [8] and the use of VDs in animal farms and imported food control [9,10]. Despite these regulations, some inappropriate use does persist: monitoring data on VD residues show that, in the EU member states of Iceland and Norway, the share of non-compliant samples of foodstuffs of animal origin ranged from 0.25–0.37% in 2009–2019, and accounted for 0.19% in 2020 [11]. In countries of the Caucasus area, animal farming is important for both food security and socio-economic development; however, regulation and control of VD are still in the beginning phase. In Armenia, the presence of illegal VD, such as residual malachite green in locally farmed fish, has been identified [12]. This finding has prompted further investigation. 

Among the VDs, nitrofurans have been widely used as broad-spectrum antimicrobials in livestock farming. However, the use of nitrofurans in food-producing animals is not authorized by the European Union (Council Regulation (EEC) No 2377/90) [13] as they can pose a serious hazard to consumers. Nitrofurans break down rapidly in the organism, and their metabolites bind to proteins, including those in muscle tissue, and can persist there for several weeks [14,15]. Nitrofurans have four persistent metabolites (NM): 3-amino-2-oxazolidinone (AOZ), 3-amino-5-methylmorpholino-2-oxazolidinone (AMOZ), 1-aminohydantoin (AHD), and semicarbazide (SEM) [16]. AOZ is a marker metabolite for furazolidone, AMOZ—for furaltadone, AHD—for nitrofurantoin, and SEM—for nitrofurazone [17,18]. Out of these four metabolites, SEM alone is found not only in animal-based foodstuffs but also in other foods [14] when using disinfectants, processing food, as well as from chemical reactions between some agents [2,14,19,20]. SEM may also occur naturally in the shells of marine arthropods [4,20]. The factual use of nitrofurans in food-producing animals is proved by protein-bound metabolites, whereas free metabolites may indicate a different residue origin [14]. Yet, to date, no research has been done to verify reliable methods of identifying the causes of NM occurrence [20]. However, it is known that, after the breakdown in the organism, NMs can move through the food chain and be excreted into the environment [21,22]. This is the reason for the presence of NMs, for example, in honey—while honeybees are not directly treated with these compounds [18].

Robust evidence supports the high toxic hazard posed by nitrofurans. Specifically, nitrofurans have been found to possess carcinogenic, genotoxic/mutagenic, and hepatotoxic potential [14,23,24]. Due to clear-cut genotoxicity and mutagenicity, a safe level of intake of residues cannot be determined; hence, risk assessment of residues resulting from illicit use or other causes is performed using the margin of exposure (MOE) approach [14]. Additionally, aside from direct toxicity, negative consequences include the enhanced development of nitrofuran-resistant strains of pathogens that are hazardous to humans as well [25,26]. 

In Armenia, regulatory documents [9,27] prohibit the presence of nitrofuran residues in fish and honey. Consequently, products with residues are unsuitable for both domestic and international trade.

In 2018, the export of fish and honey from Armenia was estimated to be USD 23,806.72 and 259.4 thousand, respectively. In 2019, fish exports increased to USD 26,894.40 thousand, while honey exports decreased to USD 158.1 thousand. In 2020, the total export from Armenia accounted for USD 2537 mln, with fish and fish-based products still increasing and accounting for 10,430.49 tons valued at USD 49,684.96 thousand (about 1.9% of the overall export), while the export of natural honey showed a remarkable decrease (20.2 tons valued at USD 136.8 thousand) [28]. Despite a challenging political situation and the pandemic, Armenia achieved more than a twofold increase in fish-based product exports over three years. However, most of the fish production is consumed within the domestic market, and about 20% of all farmed fish is exported [29]. Meanwhile, honey exports significantly recovered in 2021, amounting to USD 3289 [30]. Therefore, fish and honey are significant commodities in both domestic and international trade for Armenia. The study aims to identify NMs as the marker residues for nitrofurans in fish and honey produced in Armenia and assess the potential health risks associated with consuming these foods. This pilot study stands as the first of its kind not only in Armenia but also in the broader Caucasus region, utilizing data from an annual monitoring program to assess consumer health risks posed by NMs.

## 2. Materials and Methods

### 2.1. Survey and Data Processing

Food consumption data were obtained through a survey using the food frequency questionnaire (FFQ) method. The FFQ questionnaire featured detailed inquiries about the serving sizes, as well as the frequency of fish and honey consumption. Additionally, the questionnaire included inquiries related to food sources and demographics, including gender, age, employment, education, and income level. To enhance data precision, the portion size was formulated as an open-ended question, offering responses in grams, units, and pieces. Consumption frequency was assessed across different categories from “no consumption” to “2–4 times per week”, also including less frequent options like “2–3 per month”, “once a month”, and “other”. To calculate the average daily intake of foods, we multiplied its daily consumption frequency by the portion size and quantity.

In-person interview-based survey was conducted anonymously, ensuring a representation from all districts of Yerevan, the capital of Armenia, with 1040 respondents aged between 18 to 65 years old. Yerevan comprises about 30% of the country’s population, and the sample population is considered as representative of the Armenian adult consumers [12].

For data analysis, the statistical software SPPP (version 22.0) was used. Employing the k-means clustering method, homogeneous clusters of consumers were identified. This approach proved effective, enabling accurate calculation of the risk associated with each consumer group. Indeed, the clusters of fish consumers were identified in a previous paper [12], and we applied the same methodology to determine the clusters of honey consumers in this study.

### 2.2. Analysis of Samples

Honey and fish analyses were carried out as part of the national monitoring program concerning residues in animal-origin products. To determine the NM residues in food items, this research focused on Armenian multifloral honey and three farmed fish species highly popular among consumers and with high export potential: the Sevan trout (*Salmo Ishkhan*), Rainbow trout (*Oncorhynchus mykiss*), and Sturgeon (*Acipenserruthenus, Acipenser baerii*). The honey and fish analyses were carried out as part of the national monitoring program on residues in animal origin products, performed according to the requirements of the Council Directive 96/23/EC [31]. According to this directive, sample sizes depend on the production (in tonnes) of honey and farmed fish. For monitoring purposes, nitrofurans are included in group B2 within group B—“Veterinary medicinal products and contaminants”: for group B2, the number of tested samples should be 2/3 of the total number of samples collected [31]. In this pilot study, 9 samples of multifloral honey were collected from different domestic honey producers. A total of 15 fish specimens were procured from food stores, markets, and caught from ponds of major fish farms across different regions of Armenia.

The laboratory-based studies were conducted at the “Republican Veterinary-Sanitary and Phytosanitary Laboratory Services Center”, a State non-commercial organization. The initial screening of NM residues was done using the ELISA method. Data readout was performed by a BioTek ELx800 analyzer. To ensure accuracy, the positive results were further verified using the LQ-MS/MS method, as required by international standards [32]. Sample preparation and sample analysis were carried out in accordance with the respective test kits’ protocols and MaxSignal instructions, which included the Furazolidone (AOZ) ELISA Test Kit, AMOZ ELISA Test Kit, SEM ELISA Test Kit, and AHD ELISA Test Kit. In the case of fish, prior to the sample homogenization process, the skin and bones were removed; the muscle was grounded with a ceramic mortar.

Nitrofuran metabolites were hydrolyzed in an acidic solution and derivatized to nitrobenzyl- (NB) derivatives with 2-nitrobenzyladehyde (2-NBA). The following chemicals/reagents were used: AOZ and SEM•HCl (Sigma-Aldrich, St. Louis, MO, USA); 2-NBA (Sigma-Aldrich); DMSO (Sigma-Aldrich); d_4_-AMOZ and d_5_-AMOZ (Cambridge Isotope Laboratory, Andover, MA, USA); 1-Amino-imidazolidin-2,4-dione-[2,4,5^−13^C] (WITEGA Laboratorien Berlin-Adlershof GmbH, Berlin, Germany); Semicarbazide hydrochloride^−13^C, ^15^N_2_ (WITEGA); Ammonium Acetate (NH_4_Ac), K_2_HPO_4_, and NaOH (Sigma-Aldrich); Methanol (HPLC grade, Thermo Fisher Scientific, Pittsburgh, PA, USA) and water (in-house distilled water, filtered with a 0.45 µm filter). 

Calibration curves demonstrated linearity within a range of 0.025 ng/mL to 10 ng/mL, with R^2^ values exceeding 0.995. Rigorous validation of the method’s accuracy and precision was achieved by analyzing fish samples fortified with different concentrations of nitrofuran metabolites. The results showed recovery values ranging from 78% to 112%, along with deviations between 3% and 23%. Importantly, these standard deviations account for variations associated with both sample preparation and analytical instrument usage. The limit of detection (LOD) was equal to 0.05 μg/kg. 

### 2.3. Calculations

The risk assessment used the margin of exposure (MOE) approach. For genotoxic and carcinogenic agents, the MOE index is not considered a concern from the public health perspective when it is equal to or greater than 10,000 [14]. MOE is calculated using Equation (1):

(1) $$\mathrm{MOE}=\frac{\mathrm{BMDL}}{\mathrm{DI}}$$

where BMDL is a benchmark dose lower confidence limit (mg/kg body weight); DI—daily intake of nitrofuran metabolites (mg/kg/day). As SEM is not a genotoxic agent, the Health Based Guidance Value (HBGV) derived by EFSA was selected [14]. The HBGV and BMDL values for NM (see below Section 3.4) were derived from toxicological on animals [14]. 

The DI is calculated for each consumer cluster using Equation (2):

(2) $$\mathrm{DI}=\frac{C_{\mathrm{food}}\times C_{\mathrm{metabolite}}}{\mathrm{BW}}$$

where C_food_ (Consumption) is the average daily intake of food (kg/day); C_metabolite_ (Content)—the mean content of a metabolite in food (mg/kg); BW—a consumer’s body weight assumed as 65 kg on average.

The samples with a value exceeding the limit of detection (LOD) increase the probability of the presence of small quantities of the agent in other samples with no detectable residues; accordingly, values < LOD, “left-censored” are considered as LOD/2 to take into account the uncertainty on the presence of residues below LOD [33].

### 2.4. The Worst-Case Scenario

In risk assessment, the worst-case scenario involves considering the most unfavorable conditions or factors that could potentially occur. In this study, we have adopted a hypothetical worst-case scenario, where we consider the highest detected value per metabolite as the mean content of metabolites. Furthermore, for calculating the daily intake, we took the consumption data of the high consumers, representing the consumer cluster characterized by the highest product consumption.

## 3. Results and Discussion 

### 3.1. Consumption of Fish and Honey

According to survey data, 83% of respondents have fish in their diet; besides, fish is among the special foods commonly served during fest events. The role of occasional high intake during festive events can only be discussed after assessing regular consumption. The share of honey consumers is 70%. Among fish consumers, the statistical data analysis has revealed 3 clusters of daily intake levels. The first cluster is characterized by an average daily intake of 16 g and accounts for 79% of respondents; the second cluster consumes 63 g of fish per day and accounts for 16% of respondents; the daily diet of the third cluster—5% of respondents—includes 156 g of fish.

**Figure 1:**
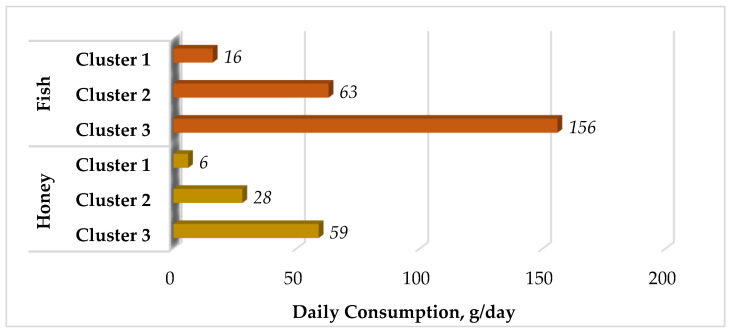
The average daily fish consumption [12] and honey among consumer clusters.

The honey consumers are also grouped into three clusters: 6 g/day, 28 g/day, and 59 g/day (Figure 1), which make up 80%, 15%, and 5%, respectively.

### 3.2. Nitrofuran Metabolites (NM) Residues in Fish and Honey

NM residues were identified in 33.3% of fish samples and 44.4% of honey samples. Considering the left-censored samples, average NM concentrations in fish samples are presented in Table 1.

Upper bound (UB) values for nitrofuran metabolites in fish and honey samples are presented in Table 2. The UB values are used to calculate the worst-case scenario.

The up-to-date reference point for action (RPA) for the four nitrofuran metabolites is 0.0005 mg/kg [20]. Any food product of animal origin found to contain NM (as single substances or as a sum) at concentrations exceeding the RPA is prohibited for sale. Conversely, if the concentration is equal to or less than the RPA, the product is marketable, but further investigation is required to determine the reasons behind the presence of NM residues [34]. In our study, 13.3% of fish samples and 33.3% of honey samples had residues above the RPA for NM. 

According to research data for 2008–2010 in Bangladesh, an unintentional source of NM in shrimps was feed [4]. A collation can be made between NM concentrations (a) determined by the Bangladeshi study, (b) identified in foodstuff batches detained during the imported food safety control in the Netherlands and France, and compared with those detected in the current study. In Bangladesh, commercial feed-caused concentrations of the studied metabolites in shrimps were as follows: SEM 1.8–3.8 μg/kg, AHD 2.92–11.29 μg/kg, AOZ 2.52–3.37 μg/kg. In shrimps fed on farm feed and fertilizers, 20% of the samples had detectable residues, and NM concentrations were as follows: SEM 1.7–4.5 μg/kg, AHD 0.93–5.8 μg/kg, AOZ 11.30 μg/kg [4]. In March 2020, the customs inspection in France registered a high concentration of AOZ (1.26 μg/kg) in frozen shrimps from India. In July of the same year, in the Netherlands, the AOZ concentration in the detained batch of shrimps imported from India was 4.7 μg/kg. In 2021, a batch of frog legs imported to France from Vietnam showed an AOZ level of 27 (+/−6.9) μg/kg (RASFF) [35]. This value suggests that residues were caused by furazolidone treatment of the animals. All of these concentrations exceed the values observed in our research—from 1.8 (AHD) to 84.4 (AOZ) times.

### 3.3. Average Daily Intake (DI) of Nitrofuran Metabolites

The EFSA Scientific Panel on Contaminants in the Food Chain (CONTAM) did not have sufficient data to establish a reference point for adverse effects caused by AMOZ. As a result, no further calculations were conducted for this metabolite. It was determined that AMOZ is not genotoxic in vitro, and there were no available in vivo data on its genotoxicity or carcinogenicity at the time of the Opinion [14]. Due to this lack of data, AMOZ was excluded from the health risk assessment.

Table 3 presents the daily intake (DI) of nitrofuran metabolites in fish and honey categorized by the “product-cluster” sector. Identifying nitrofuran residues in fish and honey samples is a framework for the possible illicit use of these compounds in Armenian agrifarming practices. Nitrofurans are banned for use in food-producing animals due to their potential health risks to consumers. The presence of these residues not only raises concerns about compliance with international food safety standards but also points toward regulatory challenges within the veterinary and agricultural sectors.

### 3.4. Margin of Exposure (MOE) for Nitrofuran Metabolites

The CONTAM Panel of EFSA has established two reference points for adverse effects related to AOZ: BMDL_10_ (and BMDL_05_, i.e., the lowest dose with a 95% probability of producing ≤ 10% or ≤5% frequency of an adverse effect, respectively. BMDL_10_ and BMND_05_ are used for quantal and continuous data, respectively, as per the EFSA approach [14]. For AHD, the BMDL_10_ corresponded to a daily dose of 29.5 mg/kg b.w. based on the induction of osteosarcoma in male rats [14]. For AOZ, the BMDL_10(neoplastic)_ corresponded to 1.6mg/kg b.w based on bronchial adenocarcinoma in mice. Additionally, a BMDL_05(non-neoplastic)_ value of 0.02 mg/kg b.w. was selected based on adverse changes in alkaline phosphatase in dogs [14]. Furthermore, SEM altered the development of bones in rats with a BMDL_10_ of 1 mg/kg b.w; this value is chosen as HBGV for the risk characterization of noncarcinogenic effects [14]. For each cluster of consumers, the MOE of AOZ was calculated in two variants: for carcinogenic effects (MOE_neoplastic_) and noncarcinogenic effects (MOE_non-neoplastic_). The calculated MOE values for fish and honey are given in Table 4 and Table 5.

In the third cluster, AOZ has the lowest MOE values: 3.47 × 10^4^ for fish and 6.89 × 10^4^ for honey. Conversely, in the first cluster, AHD has the highest MOE values: 6.31 × 10^8^ for fish and 6.15 × 10^8^ for honey. 

To assess the MOE value for the cumulative consumption of honey and fish in clusters with the highest consumption, we consider the maximum consumption values (Table 6).

It is important to note that the representatives of the third cluster for fish consumption and the third cluster for honey consumption are not the same individuals; we only consider the maximum consumption for the calculation to simulate a potential situation.

The MOE values at the sum of the maximum portions of fish and honey fall within the range of 1.04 × 10^5^ to 1.27 × 10^8^. This range represents the margin of exposure for individuals with the highest cumulative consumption of both fish and honey products. 

Using the margin of exposure (MOE) approach provides a comprehensive understanding of the potential risks associated with consuming NM-contaminated products. When the calculated MOE values exceed the established safety thresholds (i.e., MOE > 10,000 for genotoxic carcinogens), the health concerns related to genotoxicity, carcinogenicity, and other hazards are insignificant.

In the current study, the MOE values of all the studied metabolites are greater than 10,000, indicating that none of the indices oversteps the safety margin for carcinogenic as well as noncarcinogenic effects. This is observed, even with the cumulative consumption of the maximum portions of fish and honey, suggesting that occasional festive fish dishes, as well as honey, may not lead to significant health concerns.

It is worth noting that, unlike some environmental contaminants, there are no human epidemiological studies on nitrofuran (NF) metabolites. The HBGVs are solely based on animal studies, which may introduce some additional uncertainty. Nonetheless, EFSA has considered the BMDL derived from animal studies as a reliable foundation for the risk characterization of NM.

Unlike fish, honey commonly undergoes no culinary treatment before use. For this research, we sampled raw fish that did not undergo any culinary treatment before the laboratory test. NMs are known to remain almost completely preserved in food even after frying, baking, or microwaving [36]. For this reason, the authors can consider that the final results are reliably and closely representative of NM concentrations that could be encountered after cooking and consuming the fish.

### 3.5. The Worst-Case Scenario

In the worst-case scenario for fish, the highest concentrations of metabolites are as follows: AHD—1.450 μg/kg, AOZ—2.4 μg/kg, SEM—0.25 μg/kg. The value of the third cluster of consumers is assumed as C_food_, representing the largest average daily serving size. The calculation data for this scenario are provided in Table 6.

In the scenario for honey, the following concentrations of the metabolites are obtained: AHD—1.85 μg/kg, AOZ—1.93 μg/kg, SEM—0.5 μg/kg. The average daily intake is assumed to be the index of the third cluster of consumers (Table 7).

In this worst-case scenario, the MOE value for the neoplastic effect of AOZ is less than 10,000 for both fish and honey consumers. For the other two NM, the MOE values indicate no concern. In the worst-case scenario, 100% of samples have the maximum concentrations of residues. However, such samples are quite rare; for example, samples with the AOZ concentration of 2.4 μg/kg account for only 6.67% of the total number of fish samples. This indicates that such high concentrations of metabolites are not commonly found in the tested samples, further supporting the overall low risk of adverse effects associated with NM residues in fish or honey consumption in the current scenario of Armenia. Meanwhile, some uncertainties need to be considered: (a) currently, we do not have food consumption data for toddlers and children; this might lead to an underestimation of exposure, as toddlers and infants tend to eat more food per kg body weight [37]; (b) no reliable data exist to assess the health hazard of AMOZ; (c) EFSA could not consider whether NM might have additive effects, even though the target tissues seem different. Overall, these uncertainties might have lowered the conservativeness of the current health risk assessment and lend further support to the need to control the illicit use of nitrofurans in agrofarming in Armenia.

The worst-case scenario analysis employed in this study represents a crucial aspect of a risk assessment. By considering the highest detected concentrations of NMs along with maximum consumption rates, the study provides a comprehensive overview of the potential risks even under extreme, yet data-based, conditions. The fact that the MOE values remained comfortably above safety thresholds reinforces the robustness of the study’s conclusions and the safety of these food products.

## 4. Conclusions

The study showed that exposure to NM residues through consuming fish and honey produced in Armenia does not lead to significant health concerns. Meanwhile, the frequent presence of NM residues in fish flags a problem. The illicit use of nitrofurans in aquaculture does not comply with standards for domestic food marketing, and it hampers the export of food commodities to external markets. The frequent presence of NM residues, while the use of nitrofurans in honeybees is very unlikely, indicates one more concern according to a One Health perspective: the illicit use of nitrofurans leads to the potential transfer of NM into the surrounding environment, with contamination of other food chains and influence on the ecology and resistance of microorganisms. A noteworthy dimension of this study is its alignment with the “One Health” approach. The interconnectedness of human health, animal health, and the environment becomes evident in the context of illicit nitrofuran use. The residues not only affect consumer’s health but also pose risks to other animal species. This underscores the need for collaborative efforts between veterinary, agricultural, public health, and environmental sectors to address these multifaceted challenges.

Although we could not definitively identify the cause of NM occurrence in domestic fish and honey in this research, several potential reasons could be suggested: treatments of farmed animals, the illicit use of NFs as feed additives, and the use of NM-contaminated fertilizers in agriculture. Consequently, it is recommended that Armenian fish and honey producers thoroughly analyze the entire food production cycle to identify and address potential sources of contamination. Our findings further support that the presence of residues of prohibited drugs is a real issue [12] and indicates the shortcomings in veterinary control in Armenia. Such shortcomings can be overcome through establishing an integrated system based on risk analysis and prioritization, enforced regulations, efficient and quality-controlled monitoring programs, and the education and training of food safety officers and farmers, as well as through public awareness transparency, accountability, and adequate resources. 

Our data point out that the use of nitrofurans in Armenia, while not of direct public health concern, is of sufficient relevance to call for actions aimed at assessment, prevention, and monitoring; our findings may prompt further studies in other countries in the Caucasus region, that share several socio-economic and environmental features with Armenia. Beyond the identification and quantification of nitrofuran metabolites in food items, the study presents a thorough evaluation of consumer intake and associated risks. Through risk assessment, worst-case scenario examination, and actionable suggestions, the study establishes a model for addressing food safety challenges within Armenia and globally.

## Figures and Tables

**Table 1:** The concentration of nitrofuran metabolites (NMs) in fish and honey.

Nitrofuran Metabolites (NMs)	NMs Concentration (Mean ± SD, μg/kg)	
	Fish	Honey
AHD	0.19 ± 0.017	0.52 ± 0.023
AMOZ	0.1 ± 0.011	0.05 ± 0.008
AOZ	0.2 ± 0.018	0.3 ± 0.024
SEM	0.1 ± 0.012	0.4 ± 0.02

*Note: SD—standard deviation*.

**Table 2:** UB values for nitrofuran metabolites (NMs) in fish and honey samples.

UB	AHD (μg/kg)	AMOZ (μg/kg)	AOZ (μg/kg)	SEM (μg/kg)
**Fish**	1.45	0.24	2.4	0.25
**Honey**	1.85	0.075	1.93	0.5

**Table 3:** The average daily (DI) intake of nitrofuran metabolites from fish and honey.

Consumers		DI		
		AHD (mg/kg)	AOZ (mg/kg)	SEM (mg/kg)
**Fish** **consumers**	Cluster 1	4.68 × 10^−8^	5.91 × 10^−8^	2.46 × 10^−8^
	Cluster 2	1.84 × 10^−7^	2.33 × 10^−7^	9.69 × 10^−8^
	Cluster 3	4.56 × 10^−7^	5.76 × 10^−7^	2.40 × 10^−7^
	Mean	9.45 × 10^−8^	1.19 × 10^−7^	4.97 × 10^−8^
**Honey** **consumers**	Cluster 1	4.80 × 10^−8^	2.95 × 10^−8^	3.78 × 10^−8^
	Cluster 2	2.24 × 10^−7^	1.38 × 10^−7^	1.77 × 10^−7^
	Cluster 3	4.72 × 10^−7^	2.90 × 10^−7^	3.72 × 10^−7^
	Mean	9.72 × 10^−8^	5.98 × 10^−8^	7.66 × 10^−8^

**Table 4:** Benchmark dose level (BMDL) and margin of exposure (MOE) of nitrofuran metabolites for fish consumers.

Fish Consumers	BMDL, mg/kg Body Weight				MOE			
	Neoplastic Effect		Non-Neoplastic Effect		Neoplastic Effect		Non-Neoplastic Effect	
	AHD	AOZ	AOZ	SEM	AHD	AOZ	AOZ	SEM
**Cluster 1**	29.5	1.6	0.02	1	6.31 × 10^8^	2.71 × 10^7^	3.39 × 10^5^	4.06 × 10^7^
**Cluster 2**					1.60 × 10^8^	6.88 × 10^6^	8.60 × 10^4^	1.03 × 10^7^
**Cluster 3**					6.47 × 10^7^	2.78 × 10^6^	3.47 × 10^4^	4.17 × 10^6^
**Mean**					3.12 × 10^8^	1.34 × 10^7^	1.68 × 10^5^	2.01 × 10^7^

**Table 5:** Benchmark dose level (BMDL) and margin of exposure (MOE) of nitrofuran metabolites for honey consumers.

Honey Consumers	BMDL, mg/kg Body weight/day				MOE			
	Neoplastic Effect		Non-Neoplastic Effect		Neoplastic Effect		Non-Neoplastic Effect	
	AHD	AOZ	AOZ	SEM	AHD	AOZ	AOZ	SEM
**Cluster 1**	29.5	1.6	0.02	1	6.15 × 10^8^	5.42 × 10^7^	6.77 × 10^5^	2.64 × 10^7^
**Cluster 2**					1.32 × 10^8^	1.16 × 10^7^	1.45 × 10^5^	5.66 × 10^6^
**Cluster 3**					6.25 × 10^7^	5.51 × 10^6^	6.89 × 10^4^	2.69 × 10^6^
**Mean**					3.03 × 10^8^	2.67 × 10^7^	3.34 × 10^5^	1.30 × 10^7^

**Table 6:** Margin of exposure (MOE) for cumulative consumption of fish and honey by third clusters.

**Cumulative Consumption of Maximum** **Portions of Fish and Honey**	**MOE**			
	**Neoplastic Effect**		**Non-Neoplastic Effect**	
	**AHD**	**AOZ**	**AOZ**	**SEM**
	1.27 × 10^8^	8.29 × 10^6^	1.04 × 10^5^	6.85 × 10^6^

**Table 7:** A worst-case scenario for nitrofuran metabolites in the case of fish and honey consumption.

Nitrofuran Metabolites and Associated Affect	Fish		Honey	
	DI	MOE	DI	MOE
AHD (neoplastic effect)	3.48 × 10^−6^	8.48 × 10^6^	1.68 × 10^−6^	1.76 × 10^7^
AOZ (neoplastic effect)	5.76 × 10^−6^	2.78 × 10^5^	1.75 × 10^−6^	9.13 × 10^5^
AOZ (non-neoplastic effect)	5.76 × 10^−6^	3.47 × 10^3^	1.75 × 10^−6^	1.14 × 10^4^
SEM (non-neoplastic effect)	6.00 × 10^−7^	1.67 × 10^6^	4.54 × 10^−7^	2.20 × 10^6^

## Data Availability

Data is contained within the article.
